# A New Variant of Combined Pulmonary Fibrosis and Emphysema From Second-Hand Smoke: A Case Report and Review of Literature

**DOI:** 10.14740/jocmr2277w

**Published:** 2015-08-23

**Authors:** Rafay Khan, Sunil Tulpule, Nneka Iroka, Shuvendu Sen, Teena Mathew, Mohammad Islam, Abdalla Yousif, Stacey Longo

**Affiliations:** a Internal Medicine Department, Raritan Bay Medical Center, 530 New Brunswick Ave., Perth Amboy, NJ 07733, USA; b Department of Pathology, Raritan Bay Medical Center, 530 New Brunswick Ave., Perth Amboy, NJ 07733, USA

**Keywords:** Emphysema, Pulmonary fibrosis, Smoker, Second-hand smoke, Lung diseases

## Abstract

The findings of combined pulmonary fibrosis along with emphysema have been increasingly recognized in the medical literature. Patients presenting with such findings are usually found to be heavy smokers or former smokers. Their presentations begin with severe respiratory distress that gets progressively worse. They are found to have low diffusion capacity (DLCO) although spirometry will show preserved lung volumes. No prior research has presented a documented case of such a fatal condition in a young person with no prior history of smoking. In this case report, we discuss the presentation, diagnosis, and management of a young 29-year-old non-smoker with increasing shortness of breath with a complicated hospital course discovered to have an abnormal variant or presentation of “combined pulmonary fibrosis and emphysema” (CPFE). As most published studies have attributed these findings as a secondary response to a history of smoking, other etiologies and risk factors have yet to be properly analyzed resulting in prolonged hospital course and often missed diagnoses.

## Introduction

Pulmonary emphysema and idiopathic pulmonary fibrosis are distinct conditions that at times can simultaneously present and have been well described in the literature. It remains undetermined whether other risk factors exist and if there is a timetable for its occurrence. The following case will present the hospital course of a young female, non-smoker, who presented with an unusually rapid presentation of this condition, which is well differentiated from many of the typical findings that have been described in combined pulmonary fibrosis and emphysema (CPFE). The association of these two entities was first described by Wiggins et al [1] in 1990 where eight heavy smokers presented with severe breathlessness and were found to have fibrosis with upper lobe emphysema on computed tomography (CT) scan, along with low diffusion capacity (DLCO), and preserved lung volumes. The combination of these two conditions has been thus described as the coexistence of upper lobe emphysema with lower lobe pulmonary fibrosis; however, in this case both findings were discovered from the biopsy of only the right lower lung along with diminished lung volumes. This brings into question, whether there is an undiscovered or a new condition, which presents differently and may be the result of another risk factor, other than smoking.

## Case Report

A 29-year-old female with no significant past medical history presented to the emergency department with complaints of shortness of breath and productive cough consisting of green phlegm. The symptoms started a month prior for which she visited her primary medical doctor and was treated for pneumonia. Her symptoms continued to persist and gradually worsen at which point she was also treated with tamiflu for influenza. Her other symptoms however continued to progress and consisted of fever, chills, cough, body aches, and shortness of breath. The patient is a non-smoker, drinks alcohol socially, and works in a pharmacy.

**Figure 1:**
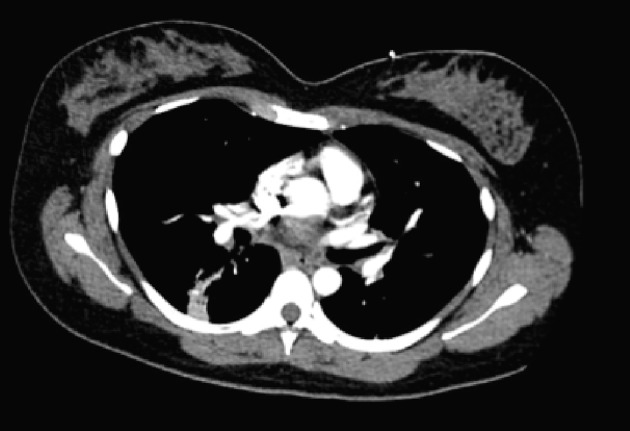
CTA chest: tree-in bud changes in right lower lobe without clear signs of emphysema.

On physical examination, the patient was tachycardia with a heart rate of 118, blood pressure of 111/72, respiratory rate of 24, and was saturating 88% on room air. At the time, she was afebrile with a temperature of 99.1 °F. Significant findings on examination showed diffuse rhonchi bilaterally with crackles on lung examination. Laboratory data demonstrated hemoglobin of 13, hematocrit of 39, white blood cell of 10.9, and platelets of 344,000. Sodium was 136, potassium 3.8, chloride 98, bicarbonate 21, blood urea nitrogen 15, creatinine 1.0, and glucose of 113. Troponins were negative. D-dimer was elevated at 0.71 and CT angiogram (CTA) was conducted to rule out pulmonary embolism. CTA of the chest was negative for pulmonary embolism but showed tree-in bud changes in the right upper lobe suspicious for an infectious or inflammatory process (Fig. 1). Similar findings were also seen in the right lower lobe. There were linear changes in the lower lobes bilaterally which most likely represented atelectasis and there was a 7.6 mm nodule in the left lower lobe.

**Figure 2:**
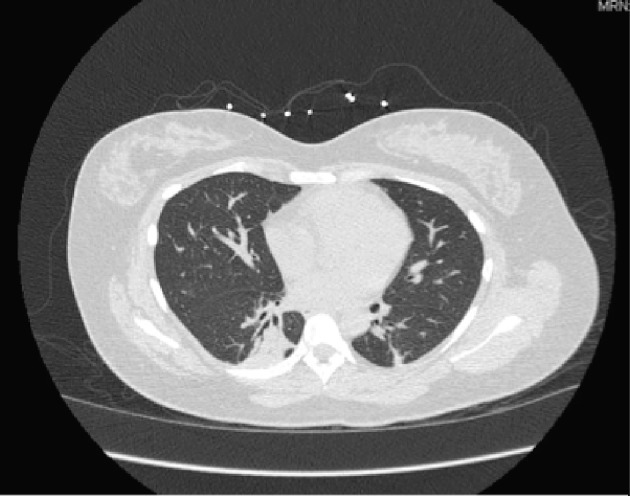
Chest CT without contrast: consolidative changes of lower lobes.

The patient was admitted to telemetry for the management of pneumonia with azithromycin and ceftriaxone initially. Patient was found to be positive for *Mycoplasma pneumonia* and medications were adjusted accordingly. Patient continued to have persistent hypoxia and continued to desaturate to 88% upon ambulation. She was later found to have hemophilus influenza growth in sputum culture, which was properly being managed by her antibiotic regimen. During her hospital stay, although the patient was receiving solumedrol and antibiotics she continued to have diffuse bilateral crackles and productive cough. Patient was found to have leukocytosis as high as 19.6, but this could be attributed to her steroid treatment. However, this train of thought remained unclear as the patient was also found to have a bandemia of 21. Her antibiotics for methicillin-resistant *Staphylococcus aureus* (MRSA) were adjusted to consist of doxycycline, zyvox, and fluconazole. The patient continued to deteriorate and systemic workup including allergy test, antineutrophil cytoplasmic autoantibody (cANCA), perinuclear anti-neutrophil cytoplasmic antibodies (pANCA), antinuclear antibody (ANA), purified protein derivative (PPD), QuantiFERON, and human immunodeficiency virus (HIV) test was all conducted. Full systemic workup returned negative for any findings. After completing her course of antibiotics, she was also removed from her steroids but would continue to desaturate and lung findings remained unchanged. Patient underwent bronchoscopy but no significant mass or lesion was found to biopsy and her postoperative diagnosis remained as non-resolving pneumonia. Repeat CT scan was performed which showed minimal improvement and continue to suggest pneumonia and possibly underlying interstitial lung disease (Fig. 2).

**Figure 3:**
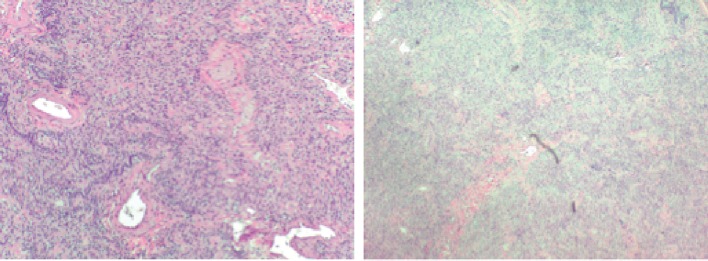
Pathological imaging suggestive of fibrotic changes.

**Figure 4:**
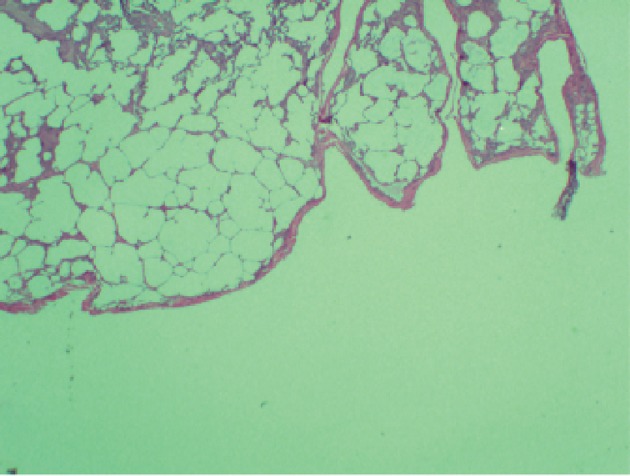
Pathological findings with questionability of emphysematous changes with alveolar septal destruction.

The patient was scheduled for video-assisted thoracoscopic surgery (VATS) procedure with biopsy of the right middle and lower lobes. Right lower lobe wedge biopsy on pathology showed lung parenchyma with consolidation, atelectasis, and areas of fibrosis (Fig. 3). Right mid lung biopsy also showed foci of consolidation but also consisted of varying stages of questionable emphysema (Fig. 4). As the findings were highly unusual and emphysematous changes were not found on patient’s CT scans, the pathology was sent to a specialist, who confirmed the results but again noted them to be very non-specific. The patient’s post-operative status was complicated and patient desaturated overnight and required intubation. Alpha-1 antitrypsin was sent but returned elevated. The patient was successfully extubated after 2 days. She was placed back on steroids and was clinically improving over the course of another 1 week. Her pulse oximetry however on room air remained around 86% even after a week of steroids and thus the patient would be discharged on home oxygen therapy. Pulmonary function tests were significant for a forced vital capacity (FVC) moderately reduced at 1.65 L (60%), forced expiratory volume in 1 second (FEV1) severely reduced at 0.98 L (40%), FEV1/FVC ratio reduced at 59%, and MVV severely reduced at 28%. Flow volume loop demonstrated a combined obstructive and restrictive contour (Fig. 4). Lung volumes showed a vital capacity reduced at 1.65 L (60%), total lung capacity (TLC) reduced at 2.08 L (56%), and residual volume (RV) reduced at 0.43 L (43%). Her diffusion capacity (DLCO) was also severely reduced at 38%. These findings were suggestive of combined severe, irreversible obstructive and restrictive lung disease with severely reduced diffusion capacity. She would follow up with infectious disease and a pulmonary specialist as an outpatient for further workup and repeat CT scan in 3 weeks with further discussion with regard to possible lung transplant options.

## Discussion

Forty years ago, prior to the use of high resolution CT scans, the combination of these two processes was first described. In a pathological anatomical study of 1,824 autopsy lungs, Auerbach et al [2] described this combination and suggested that smoking was responsible for its coexistence although these studies only involved animal models exposed to tobacco smoke. With the eventual use of CT scans, it was later discovered that patients at times would present with areas of upper lobe emphysema and other lesions compatible with lung fibrosis.

The presence of this condition is normally found in people over the age of 65 who are active smokers or heavy ex-smokers with over a 40-pack year history [3]. Patients normally present with dyspnea on exertion and Velcro crackles at lung bases with additional symptoms such as cough, wheezing, peribuccal cyanosis, and asthenia. Pulmonary hypertension has been associated with a worse clinical prognosis with this condition [3], although such a finding was not found in our patient.

The condition is a result of destruction of the lung parenchyma with characteristic remodeling resulting in lung fibrosis. Cigarette smoking has been well documented in the literature as the main etiologic risk factor [3]. Tobacco smoke contains a mix of 4,000 chemical substances associated with a broad spectrum of diseases, in this condition it has been suggested that macrophages can accumulate and via physiopathological phenomena secondary to chronic inhalation can trigger the formation of respiratory bronchiolitis and emphysema [3, 4]. Smoking is thought to trigger upper lobe emphysematous changes in patients with interstitial fibrosis, resulting in this clinical entity [5]. However, it continues to remain uncertain whether an environmental trigger or underlying genetic susceptibility can be completely excluded. Some CPFE patients have had significant exposure to agrochemical compounds, which can result in interstitial lung disease and airway damage in normally genetically susceptible smokers [6]. Others have also described CPFE in patients with particular occupational diseases such as those exposed to talc [7, 8] and welders [8, 9]. Our patient, who is a non-smoker, states she worked at a hospital particularly the pharmacy department and played a role in delivering medications to the wards. However, this association has not been clearly linked to any particular condition and as per the patient she has had this job for only 4 - 5 years.

Genetic mutations have also been illustrated in CPFE patients, which brings into question whether other risk factors other than tobacco smoking are present. Some genetic mutations, which have been described, include a case report with a mutation in protein C surfactant gene in a 32-year-old female non-smoker [8, 10] and also in a 41-year-old male non-smoker with an ABCA3 mutation [8, 11]. As there was no family history of any particular conditions, neither of these or other genetic testing was conducted in our patient. The mutations would result in dysfunction of surfactant homeostasis and cause injury to alveolar epithelial type II cells and myofibroblast proliferation [8, 10]. Moreover, these genetic conditions bring into question whether there are other hereditary components, which can increase the risk of CPFE. In terms of our case presentation, it was later determined that the patient’s mother was a chronic and heavy smoker and the patient had been exposed to second-hand smoke since an early age. Whether second-hand smoke can result in such a detrimental condition or if an underlying genetic component along with second-hand smoke can be the cause has yet to be well researched.

What make this case presentation even more unusual are the findings found on high contrast CT imaging. As previously mentioned the normal defining factor of CPFE consists of upper lobe emphysema and lower lobe fibrosis with reduced carbon monoxide transfer (DLCO). However, on imaging of our patient there were no significant findings suggestive of emphysema. There were some findings consistent with pulmonary fibrosis located in the lower lobes of the lungs. Pathological findings were positive for emphysematous changes and fibrotic lesions located in the same dissected slice from the biopsied segment. This is particularly unusual as it has not been described before to demonstrate both findings in the lower lung field. Moreover, the typical radiological changes do not appear at the same time. Cottin et al demonstrated that roughly half of the cases of emphysema and fibrosis occurred at the same time, as seen with our patient, while 25% of cases showed fibrosis occurring 5 years after the diagnosis of emphysema [12, 13].

The imaging study of choice is initially CT scan which will show centrilobular or paraseptal emphysema in the upper lobes, reticular opacities, traction bronchiectasis, septal thickening, ground-glass opacities, and honeycombing in the lower lobes [8, 13]. Once the diagnosis of interstitial pulmonary fibrosis is made, CPFE is recognized as the distinct entity by the simultaneous presence of upper lobe emphysema, which was not properly demonstrated in our patient [5]. In other cases an actual distinguishing image can demonstrate a transition area between the region of emphysema and fibrotic change, which can make it difficult to properly interpret [3, 14]. This transition area brings into question whether the section biopsied in our patient was the reason behind the difficulty in its diagnosis and supports the suggestion made by pathology to conduct an upper lobe biopsy if necessary.

Although pulmonary hypertension is a common complication during the course of this condition, it was not present upon initial diagnosis. Repeat testing may be necessary in the future as it may arise and carries a worse prognosis. Other testing frequently implemented in the diagnosis of CPFE is pulmonary function testing. FEV1, TLC, and FVC are usually within normal ranges or slightly abnormal, unlike DLCO, which will be reduced [3]. Increased pulmonary compliance along with increased inflation with loss of elasticity in areas of emphysema will compensate for the decreased volume as a result of fibrotic changes. However, the overlapping of the two pathologies will result in a negative synergic effect on gas exchange, which results in a significant decline in DLCO [3]. DLCO was determined to best correlate with the degree of parenchymal destruction as neither FVC nor TLC can be used as parameters to monitor the disease as they do not correlate with the degree of functional impairment [3]. Again, these findings other than the reduced DLCO contraindicate what was seen in our patient. Whether this is a new variant of CPFE or a completely new or different condition remains questionable.

There is no effective treatment of this disorder. The best option is smoking cessation along with lung transplantation. As our patient was a non-smoker and found to be at an advanced stage, she was later recommended to a transplant center. Bronchodilators are frequently also prescribed to patients with a positive response in their pulmonary function tests with their use. If meeting the criteria, home oxygenation can also be implemented and may be necessary. Lung transplantation is currently the only option to significantly improve survival; however, oral corticosteroids and immunosuppressant have been considered an option in the setting of CPFE [8].

Usui et al found that in 8.9% of 1,143 patients with lung cancer, there were also findings consistent with CPFE and determined that in comparison with patients with fibrosis or emphysema alone, they had a worse survival rate [15]. It has been found that the prognosis of these patients is relatively poor, with a median survival of only 2 - 6 years; however, it remains unclear if it carries a better or worse mortality than interstitial pulmonary fibrosis alone [5, 16, 17].

CPFE is a rare clinical condition that not only pulmonologists but also all physicians should be aware of and should consider in their differential when assessing patients. For example, in patients treated with a long course of antibiotics and steroids with minimal improvement in their condition, it should be taken into account. In this case presentation unlike typical CPFE, emphysema was found in the lower lobe upon biopsy, CT scan was unremarkable for emphysematous changes, lung volume changes were present, and the patient had no history of smoking. It brings into question, whether second-hand smoke or some form of undiscovered genetic component may result in a severe variant or subtype of CPFE, which can present suddenly and has yet to be discovered.
